# Repetitive Peripheral Magnetic Stimulation as an Approach to Peripherally Stimulate the Lesioned Brain

**DOI:** 10.3390/brainsci16101017

**Published:** 2026-09-24

**Authors:** Kristin Loreen Pohl, Alexander Ritter, Farsin Hamzei

**Affiliations:** 1 Department of Neurology, Moritz Klinik Bad Klosterlausnitz, 07639 Bad Klosterlausnitz, Germany; farsin.hamzei@moritz-klinik.de; 2 Section of Neurological Rehabilitation, Clinic of Neurology, University Hospital Jena, 07747 Jena, Germany

**Keywords:** magnetic stimulation, rehabilitation, stroke, repetitive peripheral magnetic stimulation

## Abstract

**Highlights:**

**What are the main findings?**

- Current evidence from randomized controlled trials and systematic reviews suggests potential benefits of rPMS for motor recovery after stroke, while findings for spasticity and activities of daily living remain heterogeneous.
- The available evidence indicates considerable heterogeneity in stimulation protocols, patient populations, and outcome measures, limiting direct comparisons across studies.

**What are the implications of the main findings?**

- rPMS represents a promising bottom-up neuromodulatory approach, but the current evidence does not yet allow definitive conclusions regarding optimal treatment protocols or long-term effectiveness.
- Future studies should use standardized stimulation protocols, adequately powered and well-defined patient populations, and longer follow-up periods to establish the clinical role of rPMS in stroke rehabilitation.

**Abstract:**

Repetitive peripheral magnetic stimulation (rPMS) is a promising neurorehabilitative technique that aims to enhance motor recovery following stroke. rPMS induces non-invasive, pain-free muscle contractions through peripheral magnetic fields and may promote central neuroplasticity via proprioceptive afferent input. Compared with neuromuscular electrical stimulation (NMS), rPMS enables painless stimulation and deeper tissue penetration. Controlled trials suggest beneficial effects particularly on upper limb motor function, whereas findings regarding spasticity and activities of daily living remain heterogeneous. Recent studies have also investigated the combination of rPMS with central stimulation techniques, such as repetitive transcranial magnetic stimulation (rTMS), as a potential approach to target both peripheral and central networks. Despite these promising findings, the generalizability and clinical implementation of rPMS are limited by small sample sizes, heterogeneous stimulation protocols, and insufficient long-term follow-up. Furthermore, rPMS is rarely included in clinical guidelines, and equipment costs may limit its widespread use. Future research should focus on adequately powered, high-quality RCTs with standardized protocols and long-term assessments to identify patients most likely to benefit from rPMS and to determine whether combinations with central neuromodulation provide additional clinical benefits.

## 1. Introduction

The implementation of effective therapeutic approaches and technologies in neurorehabilitation is of enormous socioeconomic importance. This is due to the fact that the number of people with functional limitations after a stroke is both increasing at present and is predicted to increase further in the future [1]. The development of novel neurorehabilitative therapy methods is therefore of significant importance. This development should be based on rigorous scientific evaluation to ensure the efficacy of the methods employed. In this particular context, active training-based methods are of considerable significance, given that they incorporate principles of motor learning. These principles include repetition, positive feedback and training at the limit of performance (shaping). Therapeutic measures encompass electromechanical gait training [2], treadmill training [3,4], balance, strength and endurance training [5,6,7]. These measures are employed to reduce mobility deficits and enhance participation in daily life. In the field of upper extremity rehabilitation, a range of evidence-based approaches has been identified as effective in addressing functional deficits, including constraint-induced movement therapy (CIMT), mirror therapy, and mental and task-oriented training. These methods have been found to be efficacious in improving function in upper extremity cases [8,9,10,11,12,13,14].

These therapeutic methods adopt a variety of approaches with the objective of supporting neuroplastic processes, thereby facilitating functional improvement. Nevertheless, the restoration and regeneration of upper limb functionality represent a considerable challenge, as patients with severe limitations in particular only benefit to a limited extent. In an effort to achieve further improvements, non-invasive brain stimulation methods are increasingly being used in combination with active therapies to enhance functional improvement. Non-invasive brain stimulation can be categorized into central and peripheral stimulation techniques. In contrast to central non-invasive brain stimulation approaches, which directly modulate cortical excitability through top-down mechanisms, peripheral stimulation targets muscles or peripheral nerves and may influence central motor networks indirectly through ascending afferent input. Thus, rPMS represents an emerging bottom-up neuromodulatory approach that links peripheral muscular stimulation with central neuroplastic processes.

The purpose of this paper is to provide a comprehensive overview of one of these stimulation approaches. Repetitive peripheral magnetic stimulation (rPMS) is an approach that uses a magnetic stimulation coil and aims to influence central neural processes through peripheral stimulation.

RPMS can be applied to stimulate peripheral nerves [15,16], muscles [17,18,19], or spinal roots (cervical, thoracic, and lumbar) [20,21,22,23,24]. An increase in cortical excitability has already been demonstrated following various rPMS applications [20,25,26,27].

The present review will focus primarily on rPMS applied to muscles. Repetitive peripheral magnetic stimulation (rPMS) applies rapidly changing magnetic fields to induce repeated contractions in skeletal muscles. A magnetic stimulator generates electrical pulses that are transmitted to a stimulation coil, producing a magnetic field that penetrates biological tissue and induces an electric field. This leads to depolarization and the generation of action potentials, resulting in visible muscle contractions.

### 1.1. Neurophysiological Mechanisms of rPMS

Mechanistically, rPMS is thought to operate according to a bottom-up principle, whereby stimulation originates in the periphery and is transmitted via afferent pathways to spinal and supraspinal structures. This afferent influx, primarily mediated by proprioceptive input, is considered a key driver of central neuroplastic processes. In line with this, increased activation of ipsilesional superior posterior parietal and premotor areas has been demonstrated following rPMS [27]. Similarly, neuromuscular electrical stimulation has been shown to modulate activity in the ipsilesional sensorimotor cortex, suggesting that different forms of peripheral stimulation may differentially target specific cortical regions [28]. The prevailing hypothesis, originally proposed by Struppler, suggests that rPMS enhances proprioceptive input through both indirect activation of mechanoreceptors via muscle contractions and direct excitation of afferent nerve fibers [27]. Within this proposed mechanism, afferent signals are transmitted through orthodromic and antidromic pathways [29] and are integrated within spinal and supraspinal circuits, including cortico-subcortical networks [25,26,30,31,32,33,34,35,36]. As a result, rPMS has been associated with increased cortical excitability and modulation of sensorimotor networks [20,25,26,31].

In this context, cortical excitability has been proposed as a physiological marker of neuroplastic responsiveness. Studies using non-invasive brain stimulation have conceptualized cortical excitability, commonly assessed via motor-evoked potentials, as a state-sensitive indicator reflecting the capacity of neural systems to undergo plastic changes. For example, recent work has demonstrated that modulation of cortical excitability captures within-subject changes in neurophysiological responsiveness and reflects network-level plasticity rather than purely local effects [37,38]. While these approaches are based on top-down stimulation, they support the concept that externally induced changes in neural excitability represent a link between stimulation and functional reorganization. Accordingly, rPMS-induced proprioceptive input may similarly contribute to central neuroplastic adaptations via modulation of cortical excitability and network dynamics.

Increased beta activity in the anterior cingulate cortex has been observed following combined rPMS and motor imagery, suggesting facilitation of motor imagery-related neural processes [34]. Moreover, rPMS delivered at 25 Hz has consistently been shown to enhance corticospinal excitability and reduce intracortical inhibition, whereas lower frequencies such as 10 Hz do not appear to induce comparable effects [26,31].

At the spinal level, findings remain heterogeneous. Some studies reported no significant changes in H-reflex excitability despite alterations in M-wave characteristics, indicating potential effects on neuromuscular transmission [30,32]. In contrast, increased muscle activity and reduced reciprocal inhibition have been observed following high-intensity rPMS [39], whereas other studies demonstrated reduced H-reflex amplitudes, likely mediated by activation of Ia afferents and subsequent modulation of spinal motor neuron excitability [33]. Similarly, decreased spinal reflex activity has been reported after 5 Hz rPMS, although these effects appeared less pronounced compared to tapotement massage [35,36]. Taken together, these findings suggest that the effects of rPMS at the spinal level are highly dependent on stimulation parameters such as frequency and intensity. A proposed explanation for these effects is the activation of sensory afferents, particularly Ia fibers, which may modulate spinal interneurons and reciprocal inhibition. These spinal mechanisms may represent an important interface through which peripheral stimulation influences supraspinal plasticity via ascending sensory pathways.

Figure 1 illustrates the bottom-up and top-down model.

### 1.2. Comparison Between rPMS and NMS

Given that the utilization of rPMS is frequently compared to that of neuromuscular stimulation (NMS), this comparison has become the subject of extensive academic discussion [40]. A comparison of both methods is therefore desired.

As the physical transmission of the electrical impulse differs between magnetic stimulation and NMS, which is routinely used in the rehabilitation of stroke patients [41,42,43], important differences exist between these two modalities.

A significant distinction between rPMS and electrical stimulation (NMS) is the pain-free application of rPMS. This phenomenon can be attributed to the minimal activation of cutaneous nociceptors, as the electrical field is generated exclusively in deeper tissue and muscles during magnetic stimulation. In the case of rPMS, there is an increase in depolarization of myelinated and saltatory-conducting nerve fibers. Physiologically, these fibers do not conduct nociceptive signals. This phenomenon has also been observed in fibers of groups III and IV (according to Lloyd and Hunt) [44,45]. Consequently, the generation of strong muscle contractions can be achieved without the concomitant triggering of painful sensations. Conversely, electrical stimulation involves the generation of an electrical field directly on the skin. With NMS, higher electrical stimulation intensities are necessary to induce muscle contractions or access deeper neural structures. However, this process can result in the occurrence of painful sensations. This consideration assumes particular relevance in patients diagnosed with spasticity, given that the presence of pain has been demonstrated to result in elevated muscle tone.

Furthermore, the time interval between the application of the stimulus and the response in the muscles could also be crucial. The time interval between the depolarization of motor terminal branches and the onset of the muscle twitch is estimated to be approximately 3–5 ms [46]. This temporal interval was ascertained through experimental investigation involving magnetic and electrical muscle stimulation in healthy volunteers. Electrical stimulation resulted in a wide range of latencies, ranging between 14 and 35 ms. In the group that received magnetic stimulation, the mean interval was found to be 3.5 ms. Consequently, transmission occurs more rapidly in rPMS and is associated with the physiological stimulus-reaction time. This finding could also influence the proprioceptive input enhanced by rPMS compared to electrical stimulation.

The application of rPMS differed from that of NMS in several ways. The therapeutic modality of magnetic stimulation involves the utilization of a dedicated stimulation coil, the position of which can be adjusted by the therapist between stimulation bursts. This approach facilitates the stimulation of multiple muscles during a single session, thereby enhancing proprioceptive activation. In contrast, the use of fixed electrodes in electrical stimulation makes stimulation of multiple muscles within a single session less practical. Further advantages and disadvantages of rPMS and NMS from a therapeutic perspective are summarized in Table 1.

## 2. Methods

### 2.1. Literature Search and Study Selection

This narrative review aims to summarize current evidence regarding rPMS for motor rehabilitation after stroke. A literature search was conducted in PubMed, Cochrane Library and PEDro to identify appropriate publications. This search included articles published up to May 2026 and used combinations of the following keywords: “repetitive peripheral magnetic stimulation”, “rPMS”, “peripheral magnetic stimulation”, “stroke” and “rehabilitation”.

Reference lists of relevant reviews and original articles were additionally screened to identify further studies. Randomized controlled trials, controlled clinical studies, systematic reviews, and meta-analyses investigating rPMS in stroke rehabilitation were considered for inclusion.

Studies were included if they investigated repetitive peripheral magnetic stimulation applied to muscles or peripheral nerves in individuals after stroke and reported clinical, neurophysiological, or functional outcomes. Studies focusing on other neurological conditions or non-repetitive magnetic stimulation protocols were excluded. Publications identified through the literature search and reference screening were selected based on the relevance of their titles and abstracts. Full texts were additionally reviewed when available and required to determine eligibility.

As this article is intended as a narrative overview, the selection of studies aimed to provide a representative summary of experimental and clinical evidence rather than a systematic synthesis. Accordingly, no formal risk-of-bias assessment or standardized data extraction procedure was performed. The narrative study selection may therefore be subject to selection bias and does not claim exhaustive identification of all potentially relevant studies.

### 2.2. Studies Conducted with rPMS

The first clinical trials with rPMS were initiated in the 1990s in patients following stroke or multiple sclerosis [22,47]. Recent years have seen an increase in the number of randomized controlled trials, a development which will provide a more comprehensive evaluation of the efficacy of rPMS. The following table (Table 2) provides a narrative overview of RCTs and controlled clinical trials that have been conducted on the topic of rPMS in motor rehabilitation after stroke.

The 25 included controlled trials involved a total of approximately 1127 stroke patients. Sample sizes varied considerably, ranging from 18 to 110 participants per study. The largest single study was conducted by Wang et al. (2025) [63] with 110 patients, while several studies included fewer than 40 participants.

The currently available evidence from controlled trials suggests that rPMS may promote motor recovery of the upper extremity following stroke. The vast majority of studies report significant improvements in motor function, typically measured using the Fugl-Meyer Assessment (FMA) or the Wolf Motor Function Test (WMFT) [15,16,17,23,49,50,51,53,54,55,58,59,61,63,65,66,67].

Positive effects have been described for acute, subacute, and chronic stroke patients alike. Most studies were conducted with patients in the subacute phase, although some studies included patients in various phases following stroke. Only three studies used rPMS in the acute phase [49,50,66], while Jiang et al. (2022) [54] included patients in the acute to early subacute phase.

Multiple studies have demonstrated significant improvements in spasticity as measured by the Modified Ashworth Scale (MAS) or the Modified Tardieu Scale (MTS). This has been reported by Werner et al. (2016) [19], Chen et al. (2020) [52], El Nahas et al. (2022) [56] and Aleksandrovich et al. (2023) [57], among others. However, some studies found no significant changes in spasticity, for example, Jiang et al. (2023) [15] or Fujimura et al. (2024) [60], indicating that the evidence remains heterogeneous.

Several studies also reported improvements in activities of daily living (ADL). Significant improvements in the Barthel Index (BI) or Modified Barthel Index (MBI) were described by Chen et al. (2020) [51], Jiang et al. (2022) [54], and Wu et al. (2023) [23], among others.

A notable trend in the recent literature is the combination of rPMS with brain stimulation. Studies by Yan et al. [53], Jiang et al. [15], Wu et al. [23], Qin et al. [16], Liang et al. [58], Wang et al. [63], and Yamamoto et al. [65] predominantly showed that combined protocols of rPMS and rTMS or iTBS achieved greater improvements in motor function than the respective single interventions.

However, in general the stimulation protocols used were highly heterogeneous. Frequencies ranged from 5 Hz to 50 Hz, with 10 Hz, 20 Hz, 25 Hz, and 30 Hz being the most commonly used. The number of pulses applied varied between 600 and 18,000 pulses per session. Target structures included both spastic muscles and their antagonists as well as peripheral nerve structures such as the brachial plexus, Erb’s point, the C7 root, or the nerves of the lower extremity. A plethora of studies has been conducted that has stimulated different muscle groups of the affected arm. However, there are also studies that have focused on the continuous stimulation of a single area.

The stimulation duration ranged from 15 to 30 min. In the studies reporting the number of pulses, between 600 and 18,000 stimuli were applied per session. The arithmetic mean was approximately 3455 stimuli per session, but was significantly influenced by individual high-dose protocols. The median was 1500 stimuli per session and is probably a better representation of the stimulation dose most commonly used in the literature. The majority of studies used protocols ranging from 1000 to 2400 stimuli per session.

Of the 25 studies, only four (16%) conducted long-term follow-up assessments [18,23,53,60]. The follow-up periods ranged from two weeks to three months after the completion of the intervention.

Only one study reported side effects (significant discomfort) and showed a high dropout rate (23%) in the experimental group. In this study, a combination of rTMS and rPMS was applied [63]. In other studies, no side effects were reported.

### 2.3. Systematic Reviews and Meta-Analyses of rPMS

With regard to the reviews considering rPMS, it is important to mention that all three Cochrane reviews, which demonstrated high methodological quality, found rather limited evidence for the effectiveness of rPMS [68,69,70]. However, more recent meta-analyses have identified beneficial effects on motor function and reduction in spasticity in patients after stroke [67,71,72]. Recent studies have indicated that a combination of non-invasive brain stimulation approaches may also be promising [71].

The following chart is an overview of the systematic reviews and meta-analyses, providing details of the total number of patients and the main results (Table 3).

Subsequently, the influence of rPMS on key post-stroke outcomes, including motor function, activities of daily living (ADL), and spasticity, will be discussed.

Motor function: Recent meta-analyses consistently reported significant improvements in motor function, most commonly assessed using the Fugl-Meyer Assessment for the Upper Extremity [67,71,72,75,76,77,79,80]. The magnitude of improvement ranged from mild to moderate and, in several studies, reached thresholds considered clinically meaningful. Greater treatment effects were generally observed in studies employing higher stimulation intensities and longer intervention durations. Overall, the current evidence supports a beneficial effect of rPMS on post-stroke motor recovery.

Activities of daily living (ADL): Several recent reviews and meta-analyses reported significant improvements in ADL outcomes following rPMS intervention [71,72,75]. Positive effects were observed on established measures such as the Barthel Index and the Functional Independence Measure, suggesting that gains in motor function may translate into improved functional independence. However, the available evidence remains heterogeneous, and authors frequently highlight methodological differences between studies as well as the limited availability of high-quality long-term data. In contrast, Cochrane reviews and other systematic analyses did not consistently demonstrate significant improvements in ADL outcomes [68,69,70]. These discrepancies may reflect differences in study selection, sample size, and methodological quality. Consequently, the effectiveness of rPMS for improving ADL after stroke remains uncertain.

Spasticity: The evidence regarding the effectiveness of rPMS for reducing spasticity is less consistent than that for motor function. While several meta-analyses reported significant reductions in spasticity, other reviews failed to demonstrate a statistically significant advantage over control interventions [67,68,69,70,78]. Overall, the current literature suggests a potentially beneficial effect of rPMS on post-stroke spasticity; however, findings remain heterogeneous. Variations in stimulation parameters, target structures, patient characteristics, and outcome measures likely contribute to the inconsistent results observed across studies.

Taken together, the strongest evidence currently exists for improvements in motor function, whereas evidence for ADL and particularly for spasticity remains less conclusive.

### 2.4. A Critical Evaluation of Trials Conducted with rPMS

1. The available reviews and RCTs indicate a lack of large and methodologically robust trials that examine long-term effects and identify patient groups that particularly benefit from rPMS.
2. Numerous studies have revealed a marked gender imbalance, with certain neurophysiological studies examining exclusively male subjects. Research has demonstrated that a higher proportion of men suffer strokes [81,82,83]. However, women demonstrate a higher prevalence of poorer functional outcomes after stroke than men and are disproportionately underrepresented in clinical studies [84,85]. Given the well-documented health and disease disparities between the sexes [86], recent cerebrovascular evidence further indicates that sex and age may modify the association between vascular risk factors and carotid plaque instability, supporting the consideration of sex as a potentially relevant biological modifier in stroke research [87]. There is a compelling rationale for the further emphasis on gender-sensitive research, including the domain of neurorehabilitation.
3. The utilization of disparate stimulation protocols in the various studies has rendered the comparability of the findings difficult and even questionable. The studies utilized a range of frequencies, from 5 Hz to over 50 Hz, and the number of pulses varied from 300 to over 18,000. As Luo et al. [71] and Pan et al. [72] have demonstrated, the effect size is dependent on the selected stimulation frequency and intensity. Previous meta-analyses have not consistently differentiated between dissimilar stimulation protocols.
4. Another point to consider is the determination of stimulation intensity. A variety of methodologies are employed to ascertain the intensity of stimulation, including the maximum attainable stimulation intensity of the magnetic stimulator and the resultant muscle contraction. To ensure reproducibility, the method used to determine stimulation intensity should be described precisely.

The considerable variability of stimulation protocols underscores the need for greater standardization. Recent efforts, such as the Delphi-based recommendations, represent an important step toward greater consistency and transparency in rPMS research [88]. These guidelines emphasize the detailed documentation of stimulation parameters, including frequency, intensity, coil positioning, and treatment duration, thereby facilitating the reproducibility and comparability of studies and improving the methodological quality of rPMS publications. Future research should build upon these recommendations by systematically investigating dose–response relationships and identifying optimal stimulation parameters for specific patient populations and treatment goals. Such efforts can ultimately enable the development of evidence-based treatment protocols and enhance the clinical applicability of rPMS in stroke rehabilitation.

5. Furthermore, the practical implementation of stimulation varies between studies. It has been demonstrated that discrepancies can be identified not only in the stimulation protocol, but also in the area of stimulated structures, positioning, attention guidance, and the dosage of additional therapy. The coil position is, in some cases, maintained at a constant level, while in other cases it is varied [19].
6. The validity of statements regarding long-term effects is currently limited by the lack of corresponding data spanning a sufficient time period. At the present time, the number of long-term results is limited, with only a few cases extending up to a maximum of three months [18,23,53,60].
7. The effects are also uncertain in part due to the heterogeneity of patient groups, as certain studies include patients in different post-stroke phases without evaluating them separately. Other studies have included patients with a variety of underlying neurological diseases. In such cases, the identification of patient groups that benefit most from rPMS is particularly challenging.
8. The process of determining the effect of rPMS is complicated by the use of combination therapies.
9. The methodological challenges associated with sham treatment also require consideration. A substantial number of studies utilized stimulation with identical parameters, albeit at a remarkably low intensity, which is unsuccessful in eliciting any motor response. It has been demonstrated that alternative studies utilize sham coils, which generate an acoustic resonance analogous to that produced by genuine coils but do not generate a magnetic field. An alternative option is to stimulate the affected extremity behind a curtain, thus preventing the patient from guessing the group assignment. However, in all these sham conditions, patients are deprived of visual feedback. In such a scenario this factor could undoubtedly exert an influence on the effect of the stimulation. In order to ascertain the transferability of the results to everyday therapy, it is necessary to test the effectiveness under real-life rehabilitation conditions.

In the future, there is a need for sufficient numbers of patients in methodologically robust RCTs with homogeneous patient groups. Future studies should analyze acute, subacute, and chronic stroke patients separately and report stimulation protocols accurately and in accordance with the Delphi guidelines to ensure reproducibility. Long-term follow-up assessments of at least 3–6 months should be considered in future studies to provide a more comprehensive understanding of the long-term effects of stimulation. 

### 2.5. Critical Appraisal of rPMS Reviews

The extant reviews and meta-analyses demonstrate significant methodological differences. In the initial overviews, the incorporation of non-randomized studies is justifiable, given the paucity of literature on the subject of rPMS during that period.

However, given the substantial increase in randomized controlled trials, it is logical to provide an overview exclusively of research with higher methodological quality. Due to the search strategies employed, the selection of included studies cannot be fully traced in all reviews. The Physiotherapy Evidence Database (PEDro) rating is exclusively applicable to RCTs. It is difficult for rPMS trials to attain a PEDro rating of 10/10, as blinding of the therapist administering the intervention is generally not feasible. Thus, provided that all other criteria are fulfilled, a maximum score of 9/10 may be attainable. Consequently, when interpreting PEDro-based methodological quality assessments reported in systematic reviews and meta-analyses, official PEDro ratings should preferably be considered, as these are based on ratings by two trained and independent reviewers. No formal PEDro-based quality assessment of the studies included in the present narrative review was performed. A proportion of studies included in certain meta-analyses were published exclusively in Chinese. Due to the inaccessibility of certain individual studies, it is challenging to provide definitive statements regarding their methodological quality.

In the context of meta-analyses, it is customary to incorporate all studies that are available for consideration, irrespective of their actual comparability. Nonetheless, a number of studies are of particular interest with regard to the differences and similarities in stimulation protocols. In order to generate meaningful evidence, future efforts should prioritize the conduction of high-quality RCTs.

### 2.6. Reasons for the Limited Adoption of rPMS

All the aforementioned points contribute to the argument that rPMS has not yet become a part of standard rehabilitation therapy. While the scientific evidence appears encouraging, it remains insufficient to substantiate its implementation into standard therapies. Consequently, rPMS is rarely or not at all referred to in national and international guidelines. Consequently, rPMS may still be relatively unfamiliar to physicians and therapists.

The high cost of magnetic stimulators and associated accessories is also a limiting factor in terms of the device’s widespread use. It is anticipated that the industry will be capable of producing home-based devices in a cost-effective manner, such as electrical stimulation devices. It is therefore a particularly interesting point to note the differences between peripheral electrical and magnetic stimulation.

Furthermore, uncertainty remains regarding the optimal stimulation protocol, which may vary depending on the specific indication. Differences in stimulation frequency, pulse count, and session duration may contribute to variability in treatment effects.

Additionally, the extent to which particular patient groups benefit from rPMS remains uncertain, particularly with regard to the distinction between acute and chronic cases, as well as between severe and mild symptoms.

### 2.7. Future Steps Toward Clinical Implementation of rPMS

In order to implement rPMS as a new method in clinical practice, it is necessary to conduct larger, methodologically sound RCTs with long-term follow-ups. In order to facilitate effective comparison, the stimulation protocol should be consistent and comprehensively delineated. This approach will facilitate the identification of optimal indications and patient groups for rPMS. Integration into guidelines should also contribute to improved implementation as knowledge increases.

Another promising approach is the combination of central and peripheral stimulation. The combination of tDCS and rPMS represents a potentially intriguing, albeit yet unproven, approach. This combination may provide a theoretical approach to targeting multiple networks through central modulation by tDCS and peripheral proprioceptive input induced by rPMS.

### 2.8. Combination of rPMS with rTMS and tDCS

Recent studies have investigated the combination of rPMS with centrally applied non-invasive brain stimulation, such as repetitive transcranial magnetic stimulation (rTMS) [23,58,61,65,71]. However, a number of meta-analyses have failed to identify any additional benefits arising from combinations [75].

The induction of inhibitory effects on motor cortical excitability is achieved by a low-frequency (≤1 Hz) rTMS and cortical excitability is enhanced with a high-frequency (≥3 Hz) rTMS [89]. Recent studies have demonstrated that high-frequency rTMS over the primary motor cortex enhances excitability and facilitates motor learning [90,91]. The objective of both methods is to promote plastic processes and inhibit maladaptive processes [92]. A number of studies have demonstrated enhancement in motor functions following stroke, with both high- and low-frequency stimulation [92,93]. Evidence has been presented which suggests that there has been an improvement in cases of aphasia, particularly through inhibition of the right Broca homolog region (inhibitory stimulation) [94], and a concomitant improvement in cognitive deficits, attention disorders, or neglect symptoms through targeted stimulation of parietal or prefrontal regions [95].

The combination of rPMS and rTMS has been hypothesized to facilitate motor recovery by stimulating both bottom-up and top-down processes, thereby establishing a potential closed-loop mechanism [15,16,23,58,61,63,65]. The combination of both stimulation methods was performed either simultaneously [58], or sequentially [15,61]. Improvements were observed in motor function [65], spasticity [16,63] and activities of daily living [15,16].

Another combination of rPMS and transcranial direct current stimulation (tDCS) has been proposed as a further conceivable approach to promote central reorganization processes. Transcranial direct current stimulation shifts neuronal membrane polarization, with anodal tDCS (atDCS) generally increasing cortical excitability and cathodal tDCS (ctDCS) exerting inhibitory effects [96,97,98,99]. In stroke rehabilitation, tDCS has been investigated as a complementary approach to active therapy, with studies reporting improvements in motor, cognitive, and language functions [100,101,102,103,104,105,106]. However, other studies have shown no favorable effects, and meta-analyses indicate that the overall evidence remains low to moderate [107,108,109]. Considerable heterogeneity in stimulation intensity, duration, and electrode position further complicates comparisons between studies.

Conceptually, the combination of rPMS and tDCS could involve modulation of relevant central networks by tDCS, followed by additional proprioceptive input of peripheral origin through rPMS. This combination of central and peripheral stimulation may provide a theoretical framework for facilitating neuroplastic processes. However, direct clinical evidence supporting synergistic effects of combined rPMS and tDCS is currently limited, and this concept should therefore be regarded as preliminary and hypothesis-generating. Future prospective studies are required to determine whether combined rPMS and tDCS provides additional clinical benefits and to establish appropriate stimulation parameters, including intensity and the timing of simultaneous or sequential application.

## 3. Conclusions

Although a growing number of studies have investigated the clinical effects of repetitive peripheral magnetic stimulation (rPMS) in stroke rehabilitation, the overall evidence base remains heterogeneous. Several randomized controlled trials and recent meta-analyses suggest that rPMS may improve motor function and, potentially, activities of daily living, while findings regarding spasticity remain inconsistent. However, the magnitude and consistency of these effects vary considerably across studies.

Systematic reviews and meta-analyses have highlighted substantial variability in study design, stimulation parameters, outcome measures, and patient populations. Furthermore, previous Cochrane reviews concluded that the available evidence was insufficient to support definitive recommendations regarding the clinical use of rPMS after stroke. Although more recent studies have expanded the evidence base and generally report favorable outcomes, particularly regarding motor function, the overall certainty of evidence remains limited due to methodological heterogeneity and the predominance of small single-center trials. Consequently, rPMS should currently be regarded as a promising but not yet fully established neuromodulatory intervention in stroke rehabilitation. Further well-designed randomized controlled trials with standardized stimulation protocols, clearly defined outcome measures, and long-term follow-up assessments are required to better determine its clinical effectiveness and therapeutic role.

## Figures and Tables

**Figure 1:**
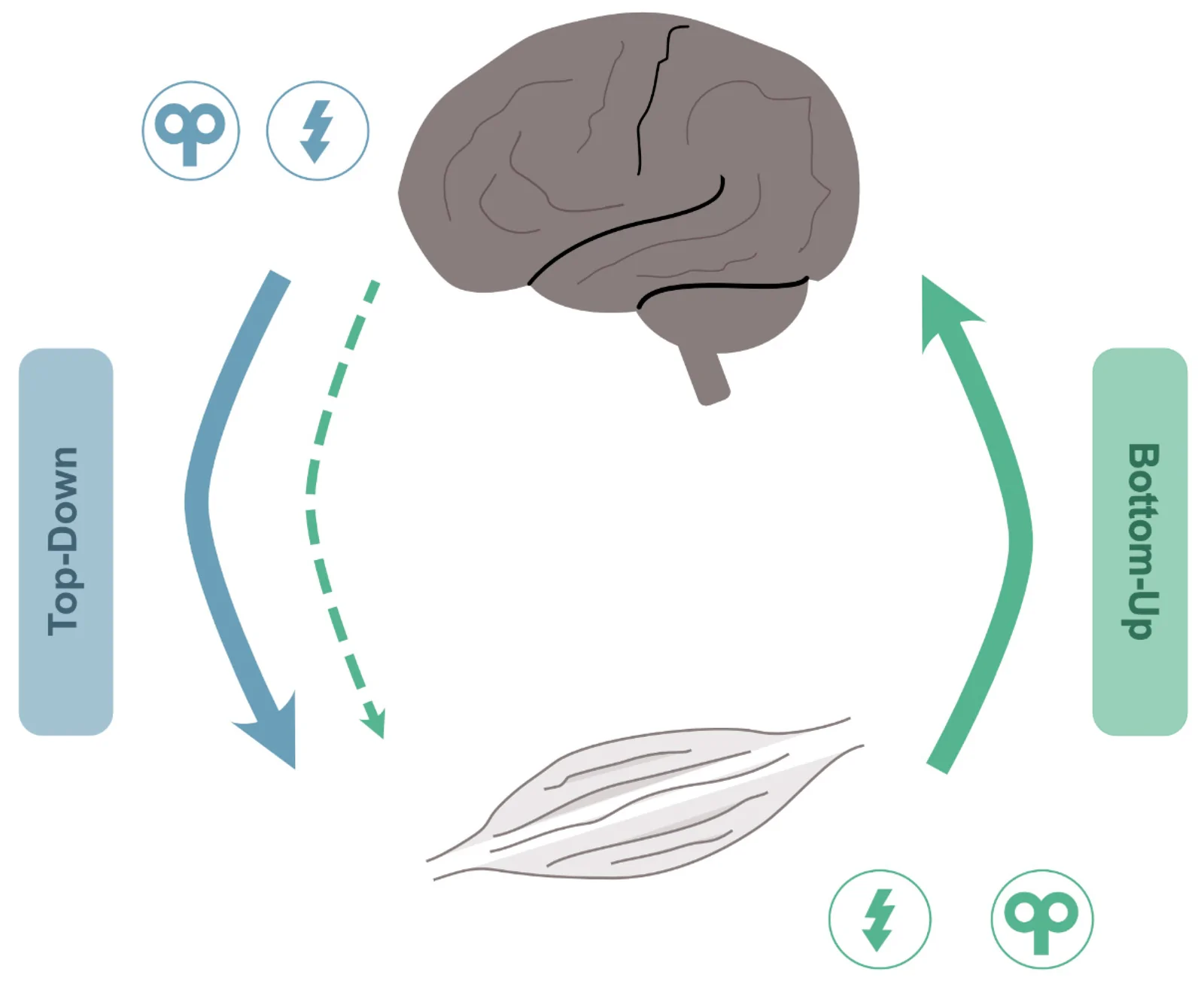
The bottom-up and top-down model. Stimulation can be delivered via magnetic or electrical stimulation directly to the brain (non-invasive brain stimulation) or to muscles or a peripheral nerve (non-invasive muscle stimulation). Repetitive peripheral magnetic stimulation can activate deep proprioceptive afferents, particularly Ia fibers, by applying a magnetic field to peripheral muscles. According to the proposed bottom-up mechanism, the resulting afferent input is transmitted via the spinal cord to supraspinal structures, including the somatosensory cortex (S1). Corticocortical connections between S1 and the primary motor cortex (M1), as well as potential antidromic activation of motor fibers, may contribute to increased cortical activation [27]. This afferent-driven bottom-up input may modulate cortical excitability and sensorimotor networks. Conversely, increased excitability within M1, accompanied by reduced intracortical inhibition, may facilitate enhanced corticospinal output. Supporting this notion, both Gallasch et al. and Nito et al. demonstrated increased MEP amplitudes following 25 Hz rPMS, together with attenuated short-interval intracortical inhibition, suggesting that afferent stimulation promotes plastic changes within motor cortical circuits. To summarize, the evidence suggests a bidirectional interaction between peripheral sensory inputs and central motor networks. Afferent stimulation may promote cortical plasticity and may enhance top-down modulation for improved motor performance and function.

**Table 1:** Advantages and disadvantages of rPMS and NMS.

	rPMS	NMS
**Mechanism of activation**	Magnetic fields induce electrical currents in nerves and muscles	Direct electrical stimulation via electrodes on the skin
**Technique**	⊕ Non-invasive skin contact via stimulation coil	⊕ Non-invasive skin contact via electrodes
**Patient comfort/tolerance**	⊕ Pain-free; no skin irritation	⊖ May be uncomfortable or painful due to irritation of the skin nerves, especially at high intensity
**Depth of stimulation**	⊕ Can stimulate deeper structures (up to several cm) and larger areas of muscles	⊖ Limited penetration depth; mainly superficial muscles
**Technical requirements**	⊖ Expensive, bulky devices; coil heating may occur	⊕ Simple, portable devices available for home use
**Use in practice**	⊖ Not yet widely used in routine clinical practice	⊕ Already widely used, particularly in physiotherapy

**Table 2:** Summary of controlled rPMS studies.

Study	Number of Patients (Total)	Stroke Phase	Protocol *(Frequency* (Hz); *Pulses; Stimulation Area)*	Outcomes	Comparison	Long-Term Follow Up	Main Results	Special Features
Krewer et al. (2014) [18]	33/33 (66)	subacute and chronic	25 Hz; 5000; extensors/flexors of the upper and lower arm	MTS; FMA; BI; HAMD-7; self-report questionnaire	Sham	2 weeks after treatment	No significant improvement/changes in FMA, BI, HAMD-7 or pain Reduction in spasticity in the MTS	
Beaulieu et al. (2015) [48]	9/9 (18)	chronic	iTBS (50 Hz); 600; tibialis anterior	ROM; isometric muscle strength; resistance of plantar flexors to stretch; TMS	Sham	No	Improvement in strength, mobility, and spasticity TMS results were inconsistent	rPMS lower extremity
Werner et al. (2016) [19]	20/20 (40)	chronic	5 Hz; 750; hand and finger flexors	MAS; ROM via goniometer MCP II-V	Sham + stretching	No	Reduction in MAS scores for finger and hand flexors	
Yang et al. (2018) [49]	15/15 (30)	acute	5 Hz; N/A; supraspinatus & deltoid	Measurement of shoulder structures using ultrasound; FMA	Electrical stimulation	No	Reduction in shoulder subluxation Increase in muscle thickness Improvement in FMA	Case–control study
Obayashi et al. (2020) [50]	10/9 (19)	acute	30 Hz; N/A; deltoid, triceps, biceps, extensor carpi radialis, flexor digitorum superficialis	FMA; WMFT; FAS; BBT	Standard therapy	No	Improvement in FMA and WMFT No significant differences in BBT	
Chen X et al. (2020) [51]	20/20 (40)	subacute	30 Hz; N/A; extensors of the upper extremities and shoulder muscles	FMA; BI	Low-frequency rTMS	No	Significantly greater improvements in FMA and BI	rPMS + low-frequency rTMS as control intervention
Chen S et al. (2020) [52]	16/16 (32)	subacute and chronic	5 Hz spastic muscles, 20 Hz antagonists; 750 spastic muscles, 5100 antagonists; shoulder abductors and extensors, elbow flexors and extensors, hand flexors and extensors	MAS, MTS; FMA; EEG; pain and changes in spasticity questionnaires	Sham	No	Reduction in MAS and MTS for elbow and hand flexors Improvement in FMA Increase in mu-rhythm activity over sensorimotor cortex	
Yan et al. (2021) [53]	18/17 (35)	subacute	10 Hz; 1200; paretic muscles of the upper and lower extremities, M1	FMA; NIHSS; MBI	rTMS + sham rPMS	4 and 12 weeks after initiation of treatment	Improvement in FMA (significant differences between groups only after stim UE 2 weeks, LE 12 weeks) No difference in NIHSS and MBI between the groups	Combination of rPMS + rTMS
Jiang et al. (2022) [54]	24/20 (44)	acute to early subacute phase	20 Hz; 2400; triceps, extensor digitorum	FMA; BI; MAS; EMG	Conventional therapy	No	Improvement in FMA, BI and muscle strength	All patients received NMS
Ke et al. (2022) [55]	15/15 (30)	subacute	20 Hz; 1800; simultaneous brachial plexus + tibial nerve/common peroneal nerve	FMA; MRC	Sham	No	Improvements in FMA and proximal muscle strength in the MRC No differences in distal muscle strength	High-frequency rPMS of axilla and poplitea simultaneously
El Nahas et al. (2022) [56]	27/15 (42)	subacute and chronic	50 Hz (iTBS); 600; biceps, finger and hand flexors	MAS; eBTD	Sham	No	Reduction in MAS eBTD reduction 35%	rPMS with iTBS (various neurological diseases)
Jiang et al. (2023) [15]	18/19/19 (56)	subacute	10 Hz; 1200; Erb’s point, affected head area	FMA; MBI; ARAT; MAS	Central iTBS	No	Combination treatment showed greatest improvements in motor functions and daily living skills No change in MAS	Combination of rPMS iTBS
Wu et al. (2023) [23]	15/15/15/15 (60)	subacute	10 Hz; 1000; cervical nerve root (C5-Th1), M1	FMA; WMFT; MBI	Only rTMS, only rPMS + sham rTMS, sham	3 months after treatment	All stimulation groups improvement in FMA, WMFT, MBI Greatest improvements in combination group Effects of the combination group still detectable after three months	Combination of rPMS on cervical nerve roots + rTMS
Fawaz et al. (2023) [17]	40/40 (80)	subacute and chronic	30 Hz; 18,000; shoulder abductors, elbow extensors, wrist extensors, supinator	FMA; ultrasound assessment; FIM; AROM; MAS	Sham	No	Improvements in FMA Reduction in MAS Improvement everyday skills in FIM Improvement range of motion in dorsiflexion and supination No significant changes in muscle cross-section (but reduction in the control group)	
Qin et al. (2023) [16]	20/15/14 (49)	subacute	10 Hz; 1200; Erb’s point, M1	MAS; FMA; MBI; fMRI	rTMS + conventional therapy	No	Combined group (rPMS + rTMS) shows strongest improvements on FMA, MAS and MBI Increased ALFF right supplementary motor area, right middle frontal gyrus and right cerebellum Reduced ALFF right postcentral gyrus	Combination of rPMS + rTMS
Aleksandrovich et al. (2023) [57]	20/20 (40)	subacute	N/A; N/A; motor points of spastic muscles and antagonistic muscles	MAS; MTS; ArmA	Sham	No	Improvement in MAS, ArmA and angles of spasticity (MTS) in both groups,	
Liang et al. (2024) [58]	15/15/15 (45)	early subacute phase	5 Hz; 1200; C7 nerve root, M1 (rTMS)	FMA; FCA; BI; SICI	HF-rTMS, standard therapy	No	Improvement in FMA, BI and FCA in rTMS and combination group Reduced intracortical inhibition in combination group	Combination of rPMS + rTMS
Yan et al. (2024) [59]	30/30 (60)	subacute	20 Hz; N/A; Erb’s point, deltoid, biceps, brachial nerve	FMA; VAS; ROM	Sham TMS and conventional therapy	No	Improvement in FMA, ROM and pain	
Fujimura et al. (2024) [60]	25/25 (50)	subacute	30 Hz; 12,000; supraspinatus, deltoid/infraspinatus	Change in AHI T0-T1 between groups; Change in AHI T0-T1; each group; NRS shoulder pain; MAS; FMA	Standard therapy	6 weeks post intervention	Subluxation in stimulation group lower Improvement in AROM in control group No difference in FMA, pain or spasticity	
Chang et al. (2024) [61]	14/14 (28)	subacute and chronic	5 Hz/50 Hz; 600 rPMS, 600 iTBS; radial groove, affected primary motor cortex	FMA; ARAT; FIM; SIS;	Sham	No	Improvement in FMA (not significant between groups) No improvement in ARAT, SIS, FIM	Combination of rPMS + iTBS
Prabhu et al. (2024) [62]	25/25 (50)	subacute	N/A; N/A; N/A	MI; BBT	NMES	No	Improvement in MI and BBT	
Wang et al. (2025) [63]	52/58 (110)	subacute	10 Hz rTMS/1 Hz rPMS; M1 rTMS/Erb’s point rPMS	MAS; FMA	Sham	No	Improvement in FMA and MAS	Combination of LF-rPMS + HF-rTMS
Fujimura et al. (2025) [64]	9/9 (18)	subacute	30 Hz; 6000; dorsum of the hand, intrinsic hand muscles	Changes in hand edema by volumetric meter; circumference of the hand; ROM; pain, numbness, grip strength	Conventional therapy	No	Reduced hand edema Increased MCP joint flexion	
Yamamoto et al. (2025) [65]	23/23/23 (69)	subacute	10 Hz rTMS/25 Hz rPMS; 1000 rTMS/1000 rPMS; M1 rTMS/forearm muscles rPMS	FMA	Conventional therapy, conventional therapy + rTMS	No	Greatest improvement in FMA in the combination group rPMS + rTMS + conventional therapy	Combination of rPMS and rTMS
Obayashi et al. (2026) [66]	9/9 (18)	acute	30 Hz; 1800; deltoid, triceps, biceps, forearm flexors, -extensors	WMFT; FAS; FMA; TLT	Conventional therapy	No	Improvement in WMFT, FMA and TLT	

Table abbreviations: *Spastic scales/measurements*: **MAS** = Modified Ashworth Scale; **MTS** = Modified Tardieu Scale; **eBTD** = Estimated Botulinum Toxin Dose; *Functional tests*: **FMA** = Fugl-Meyer Assessment; **WMFT** = Wolf Motor Function Test; **BBT** = Box and Block Test; **ARAT** = Action Research Arm Test; **MI** = Motricity Index; **ArmA** = Arm Activity Measure; **fMRI** = Functional Magnetic Resonance Imaging; **ALFF** = Amplitude of Low-Frequency Fluctuation (reflect the local spontaneous brain activity during the resting state). *Muscle strength scale*: **MRC** = Medical Research Council; *ADL scale*: **MBI** = modified Barthel Index; **FAS** = Functional Ability Scale; **FCA** = Functional Comprehensive Assessment. *Stroke Severity*: **NIHSS** = National Institutes of Health Stroke Scale; **AHI** = Acromio-Humeral Interval (*distance between the undersurface of the acromion and the top of the head of humerus*); **(A)ROM** = (Active) Range of Motion; **TLT** = Thumb Localizing Test.

**Table 3:** Summary of systematic reviews on rPMS.

Review	Type	Included Studies	Total Number of Patients	Main Results
Beaulieu et al. (2013) [73]	Review	13 (various study designs)	226	Reduction in spasticity after rPMS of the muscles or spinal roots Improvement of range and speed of movement Activation of fronto-parietal networks Reduced spinal excitability (Studies with healthy volunteers and patients with various underlying neurological diseases included)
Beaulieu et al. (2015) [74]	Review	24 (various study designs)	-	Promising effect on pain and functional disorders Parameter selection (coil, frequency, intensity, and duration) should be tailored to the treatment target
Momosaki et al. (2017) [69]	Systematic review (Cochrane)	3	121	No evidence for rPMS as standard therapy, little effect on spasticity
Sakai et al. (2019) [70]	Systematic review (Cochrane)	4	139	No significant improvement in ADL or motor skills, slight reduction in spasticity
Kamo et al. (2022) [68]	Systematic review (Cochrane)	4	139	No significant effects on ADL or motor skills, only slight reduction in spasticity
Pan et al. (2022) [72]	Meta-analysis	8	297	Significant reduction in spasticity Improvement of motor skills Improvement of ADL (various underlying neurological diseases)
Chen et al. (2023) [67]	Meta-analysis	5	188	Improvement of motor skills No significant change in spasticity
Luo et al. (2024) [71]	Meta-analysis	9	483	rPMS in combination with rTMS Improvement of motor skills and ADL Reduction of spasticity
Wang et al. (2024) [75]	Meta-analysis	17	657	rPMS alone and rPMS in combination with rTMS improves upper extremity motor function and ADL No evidence that combination of rPMS and rTMS is more effective Number of pulses per session and total pulses significant predictor of ADL effect size
Silva et al. (2024) [76]	Scoping Review	52 (various study designs) (20 rPMS Studies)	1125	Improvement FMA Reduction of spasticity
Liao et al. (2025) [77]	Meta-analysis	8	343	Improvement FMA Reduction of spasticity
Keesukphan et al. (2025) [78]	Network Meta-analysis	62 (4 rPMS Studies)	1476	Greatest effects on motor function with combined NMS and rPMS No significant superiority in spasticity reduction and ADL
Liu et al. (2025) [79]	Meta-analysis	12	492	Improvement in arm and hand function and ADL No statistically significant effect on spasticity reduction
Hui et al. (2026) [80]	Meta-analysis	14	580	Improvement FMA Reduction in spasticity Optimal stimulation parameters: frequency: 10 Hz; duration: 10–20 min; 20–55% of maximum stimulator output; 21 days of treatment; In the subacute phase, nerve stimulation was more effective than muscle stimulation

Abbreviations: **ADL** = Activities of Daily Living; **FMA** = Fugl-Meyer Assessment. **Impact of rPMS on multiple symptoms**.

## Data Availability

No new data were created or analyzed in this study. Data sharing is not applicable to this article.

## Abbreviations

AHI	Acromio-Humeral Interval
ALFF	Amplitude of Low-Frequency Fluctuation
ARAT	Action Research Arm Test
AROM	Active Range of Motion
BBT	Box and Block Test
BI	Barthel Index
CIMT	Constraint-Induced Movement Therapy
CT	Clinical Trial
ctDCS	Cathodal Transcranial Direct Current Stimulation
eBTD	estimated Botulinum Toxin Dose
EEG	Electroencephalography
EMG	Electromyography
EMS	Electrical Muscle Stimulation
FAS	Functional Ability Scale
FCA	Functional Comprehensive Assessment
FES	Functional Electrical Stimulation
FIM	Functional Independence Measure
FMA	Fugl-Meyer Assessment
GABA	Gamma-Aminobutyric Acid
HAMD-7	7-item Hamilton Depression Rating Scale
Hz	Hertz
iTBS	Intermittent Theta Burst Stimulation
LE	Lower Extremity
M1	Primary Motor Cortex
MAS	Modified Ashworth Scale
MBI	Modified Barthel Index
MEP	Motor-Evoked Potential
MI	Motricity Index
MRC	Medical Research Council
MTS	Modified Tardieu Scale
NIHSS	National Institutes of Health Stroke Scale
NMS	Neuromuscular Stimulation
PEDro	Physiotherapy Evidence Database
RCT	Randomized Controlled Trial
ROM	Range of Motion
rPMS	Repetitive Peripheral Magnetic Stimulation
rTMS	Repetitive Transcranial Magnetic Stimulation
S1	Primary Somatosensory Cortex
SICI	Short-Interval Intracortical Inhibition
SIS	Stroke Impact Scale
tDCS	Transcranial Direct Current Stimulation
TLT	Thumb Localizing Test
TMS	Transcranial Magnetic Stimulation
UE	Upper Extremity
WMFT	Wolf Motor Function Test
